# Sentinel-2-Based Forest Health Survey of ICP Forests Level I and II Plots in Hungary

**DOI:** 10.3390/jimaging11110413

**Published:** 2025-11-14

**Authors:** Tamás Molnár, Bence Bolla, Orsolya Szabó, András Koltay

**Affiliations:** 1 Forest Research Institute, Department of Forest Ecology and Silviculture, University of Sopron, Várkerület 30/A, 9600 Sárvár, Hungary; bolla.bence@uni-sopron.hu; 2 Forest Research Institute, Department of Plantation Forestry, University of Sopron, Farkassziget 3, 4150 Püspökladány, Hungary; szabo.orsolya@uni-sopron.hu; 3 Forest Research Institute, Department of Forest Protection, University of Sopron, Hegyalja út 18, 3232 Mátrafüred, Hungary; koltay.andras@uni-sopron.hu

**Keywords:** forest monitoring, satellite imagery, ICP Forests, defoliation, forest hydrology

## Abstract

Forest damage has been increasingly recorded over the past decade in both Europe and Hungary, primarily due to prolonged droughts, causing a decline in forest health. In the framework of ICP Forests, the forest damage has been monitored for decades; however, it is labour-intensive and time-consuming. Satellite-based remote sensing offers a rapid and efficient method for assessing large-scale damage events, combining the ground-based ICP Forests datasets. This study utilised cloud computing and Sentinel-2 satellite imagery to monitor forest health and detect anomalies. Standardised NDVI (Z NDVI) maps were produced for the period from 2017 to 2023 to identify disturbances in the forest. The research focused on seven active ICP Forests Level II and 78 Level I plots in Hungary. Z NDVI values were divided into five categories based on damage severity, and there was agreement between Level II field data and satellite imagery. In 2017, severe damage was caused by late frost and wind; however, the forest recovered by 2018. Another decline was observed in 2021 due to wind and in 2022 due to drought. Data from the ICP Forests Level I plots, which represent forest condition in Hungary, indicated that 80% of the monitored stands were damaged, with 30% suffering moderate damage and 15% experiencing severe damage. Z NDVI classifications aligned with the field data, showing widespread forest damage across the country.

## 1. Introduction

Forest health monitoring is crucial for understanding ecosystem conditions and responses to stress. Forests in Europe are increasingly affected by drought, insect outbreaks, wind, snow, and frost. Europe experienced a particularly severe drought from spring to autumn 2022. As the effects of the drought were felt nationwide, a large-scale national survey was necessary. In Hungary, the last decade has witnessed a notable increase in damage incidents, with prolonged droughts leading to widespread declines in forest health. The particularly severe drought in 2020 and 2022 highlighted the urgent need for efficient, large-scale monitoring methods capable of detecting disturbances in near real time. Traditional ground-based monitoring programmes such as ICP Forests [1] provide valuable insights but are often labour-intensive and spatially limited, creating demand for complementary remote sensing approaches.

The International Co-operative Programme on Assessment and Monitoring of Air Pollution Effects on Forests (ICP Forests) [2] has long tracked tree crown condition (defoliation, discolouration, etc.) through ground surveys on Level I and Level II plots [1,2]. The ICP Forests was initiated in 1985 to monitor forest conditions in forty-two countries at two levels of monitoring. Level I monitoring is based on 6000 monitoring plots arranged in a 16 × 16 km systematic transnational grid to gain insight into geographical and temporal changes in forest condition, providing annual regional surveys of crown defoliation and damage. Level II intensive monitoring includes 640 sample plots in selected forest ecosystems, which are intensive study sites with continuous measurements (growth, soil, meteorology, etc.) and detailed crown condition records.

The continuous field assessments are labour-intensive and spatially sparse, while the integration of satellite remote sensing provides continuous, large-area coverage [3]. ICP Forests datasets can be supported by remote sensing for assessing forest health by detecting various stress factors, as Solberg (2019) suggested [4]. Satellites such as Sentinel-2 and 3, Landsat 8 and 9, and MODIS offer data for monitoring defoliation, discolouration, drought, and environmental disturbances like wind and snow. Defoliation is observed through reduced vegetation indices (like Normalised Difference Vegetation Index (NDVI)) and Fraction of Absorbed Photosynthetically Active Radiation (FAPAR), both detectable using optical satellites. Discolouration, often linked to nutrient deficiencies, is tracked by reduced canopy chlorophyll levels, while LIDAR detects canopy penetration. Drought is assessed by measuring Vegetation Water Content with SMOS and detecting increased surface temperatures via Sentinel-3. Wind and snow damage are observed as reduced tree height, detectable using radar and optical sensors. This integrated remote sensing data enables efficient monitoring and early detection of forest stress, making it a powerful tool in forest management and conservation.

Sentinel-2 satellites (S2) of the European Space Agency, with their high-resolution multispectral imagery and frequent revisit (2–5 days), offer an ideal data source for forest health assessment [5]. Utilising high-resolution S2 imagery and Google Earth Engine (GEE) cloud computing, a forest monitoring method was created to detect forest disturbances remotely, swiftly, and conveniently.

Recent studies have demonstrated that Sentinel-2-derived vegetation indices and time-series anomaly detection can effectively capture changes in forest condition (e.g., drought impacts, defoliation) and complement ground observations from ICP Forests [6,7]. Ground data from both levels serves as a vital reference for satellite assessments. For example, Hungarian researchers used Level I and II plots to compare Sentinel-2 indicators with field defoliation records. They found good agreement between satellite-derived forest health indices and the ICP defoliation classes, despite timing differences [8]. Such integration enables validation of remote sensing methods and calibration of satellite indices to the ICP standards of damage assessment [9].

Extreme droughts in Europe (e.g., 2018, 2022, 2024) have offered test cases for Sentinel-2-based monitoring [10,11]. Puletti et al. (2019) evaluated multitemporal Sentinel-2 imagery in Italy to detect crown dieback after the 2017 drought, using decreases in NDVI to map areas of severe beech defoliation [12]. They confirmed that S2 can capture widespread foliage loss and subsequent partial recovery in the following year, matching field and airborne observations (e.g., full canopy recovery by summer 2018 for many stands) [13]. NDVI and NDMI trajectories were analysed from 2017–2019 to assess forest changes under bark beetle attack and post-drought recovery [14,15]. They found that NDMI was sensitive to drought-related disturbances and tree mortality—areas affected by stress had NDMI values as low as 0.1–0.2 (median), whereas healthier forests had higher index values. NDMI also distinguished the stages of bark beetle outbreak (grey stage) better than pure greenness indices. Across Europe, the hot drought in 2018 caused measurable drops in satellite greenness; for instance, Z NDVI anomaly maps in Hungary showed a negative deviation in 2018 for one-third of forest compartments, coincident with known drought damage [16]. These examples demonstrate that Sentinel-2 indices can effectively detect drought-induced defoliation.

Beyond drought, Sentinel-2 has been used to map insect damage and general crown conditions. Bárta et al. (2021) achieved early detection of bark beetle infestations in Central European spruce forests by spotting subtle declines in red-edge and infrared reflectance months before visible mortality [17]. Time-series NDVI from Sentinel-2 helped delineate outbreak progression, and combining data from consecutive Sentinel passes improved detection of small clearings from salvage logging [16]. In broadleaf forests, defoliating insects or late-spring frost events can also be captured. For instance, the Hungarian Level II analysis noted a sudden NDVI drop in 2017 at one site due to a late frost, which satellite data clearly reflected, followed by a rebound in 2018. This aligns with other reports that Sentinel-2 imagery detected short-term foliar loss due to spring frost in beech forests, as well as the recovery over subsequent growing seasons. Overall, by examining year-to-year changes, Sentinel-2 time-series enables identification of various disturbance types like drought, biotic attack, storm, or frost, which showed disorientation in vegetation indices [18]. Importantly, remote sensing analyses are increasingly validated against ICP Forests observations of defoliation and damage, ensuring that satellite-detected disturbances correspond to changes in canopy condition [8].

Multi-spectral imagery from Sentinel 2 allows computation of various vegetation indices sensitive to canopy condition. Besides NDVI, Normalised Difference Moisture Index (NDMI) measures canopy water content and is a key indicator of water stress and potential drought [19]. In healthy dense forests, NDVI and NDMI tend to be high, while stressors like defoliation, drying, or pest attack cause declines in these indices [20]. Tasselled cap indices (greenness, wetness) derived from Sentinel 2 have been tested alongside NDVI and NDMI to discriminate disturbance stages [14].

A huge advantage of Sentinel 2 is the availability of frequent observations to build time series of vegetation index values and multi-temporal data to detect deviations from normal phenology or gradual trends [3]. Anomaly detection typically involves comparing current index values to a baseline (historical mean or a reference area) and identifying significant drops. One method is computing standardised anomalies of NDVI (Z NDVI) over time. Molnár and Király (2024) created annual Z NDVI maps for the growing seasons of 2017–2023 across Hungary’s ICP Level II sites [16]. By standardising NDVI relative to the mean and variability of a healthy reference, they classified each 10 m pixel into damage classes [8]. Such Z NDVI anomaly maps proved effective for showing foliage loss due to stress.

Other studies perform time-series trend analysis or breakpoint detection: for example, identifying a sharp NDVI and NDMI decline between consecutive years as a disturbance event [14]. Overall, the full time-series improves reliability by distinguishing disturbances from phenological noise [21].

## 2. Materials and Methods

### 2.1. Study Site

**Figure 1:**
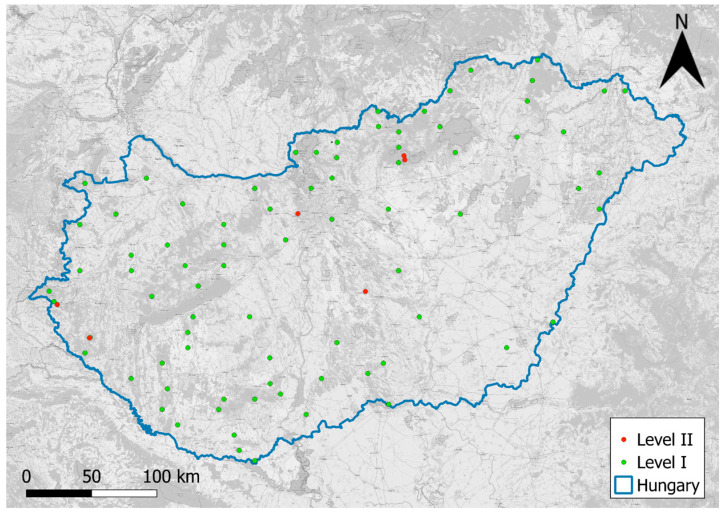
ICP Forests level I and II plot locations in Hungary.

Our study area is the entire Hungary with 78 ICP Forests Level I and 7 Level plots (Figure 1). Hungary is situated in Central Europe and has a temperate continental climate (Dfb class according to Köppen). The country receives an annual mean precipitation of 500–800 mm. Hungary enjoys 1900–2100 h of sunshine per year, especially in the southern part of the country. Around half of our country is flat, with the Great Hungarian Plain in the central and Eastern part.

### 2.2. ICP Level II Data

The Level II health surveys of ICP Forests are carried out according to a methodology applied by the European member states of ICP Forests [1,2]. The survey methodology allows a detailed and accurate determination of each tree’s location, extent, and damage triggers.

Surveys of health conditions are made twice a year. The first recordings take place between 15th May and 15th June, while the second recording is in August. The health status is recorded for a varying number of sample trees per plot, averaging 100 (minimum 50, maximum 200), depending on the stand type. This includes an assessment of crown condition and damage to the trunk and roots. Among others, the nature and extent of discolouration and defoliation, the extent, characteristics, and causes of branch and trunk damage, and root damage are recorded.

Defoliation of the crown is a key determinant of tree health. Based on the percentage of defoliation, the following categories are distinguished, where defoliation and branch dieback are summarised by weighted average but also grouped by the cause (Table 1).

In addition to recording health status, extensive meteorological data collection is carried out in the ICP level II sample areas. Of these, the amount of precipitation during the growing season and the trends in average temperature are of crucial importance for tree health. These two parameters are used to calculate the Temperature Precipitation Factor (TPF) for each year (TPF = 100 × (average temperature)/(total precipitation) for the period March-August). This value is used to characterise the weather during the most important period for tree development each year. According to this calculation, the higher the TPF value, the more unfavourable the weather was during the growing season of the year.

The sample area of the Mátra Mountains with sessile oak (*Quercus petrea*) (M03) and beech (*Fagus sylvatica*) stands (M01) showed almost identical health parameters. In both areas, a gradual deterioration of health status was observed over the last decade, which correlates with a trend increase in TPF. In addition to unfavourable climatic conditions, other factors have also played a role in the data measured in the stands in certain years. In 2017, we recorded a high leaf loss in both stands due to higher winds and, in the case of oak, a surge in powdery mildew infections. In the beech stand, wind in 2021 and drought in 2022 caused major damage to crowns. In contrast, in the oak stand, these effects caused average foliage loss. However, in 2023, increasing defoliation was observed on oaks due to the mass occurrence of the oak lace bug (*Corythucha arcuata*).

The TPF in the western part of the country (Zala County, Őrség) has remained unchanged in recent years and reflects more favourable precipitation and temperature conditions than in other parts of the country. Accordingly, the Scots pine (Pinus sylvestris) (M15) and sessile oak (*Quercus petraea*) (M16) plots show a favourable health status year to year in the Őrség, with no outstanding crown damage. The higher rates of defoliation shown in the graphs in 2013–2014 are due to the gradation of the gypsy moth (*Lymantria dispar*). With the collapse of the gradation, the stand recovered quickly.

The overall health of the trees in the beech (M17) stand in Zala is good, similar to the areas in the Őrség. In some years, minor damage events occurred, but their impact in the longer term does not yet show a trend towards deterioration. In two years, higher average defoliation was observed, in 2021 due to wind and in 2022 due to drought.

The Turkey oak (*Quercus cerris*) (M21) plot was established near Budapest in 2020; the trees are in particularly good condition, despite the TPF value being high, indicating a continuous unfavourable dry drought weather. In 2020, a minor amount of gypsy moth (Lymantria dispar) chewing was recorded in the first year of the surveys.

The most unfavourable conditions in all respects were recorded in the black locust (*Robinia pseudoacacia*) (M19) plot in the central part of the country. The health status of the trees is particularly poor and is steadily deteriorating, as evidenced by the extremely high TPF compared to the national average and the upward trend. In 2022, drought and high numbers of leaf miners led to a significant reduction in canopy cover, while in 2023, drought and the degradation processes initiated in previous years again led to higher average defoliation.

### 2.3. ICP Level I Data

The dataset comprises field measurements from 78 ICP Forests Level I monitoring plots, a total of 1644 trees. This includes annual records across 78 plots over seven years, with key health indicators recorded: defoliation percentage, crown dieback percentage, and discolouration. These parameters provide insight into the physiological condition and stress levels of the trees.

### 2.4. Satellite Imagery and Cloud Processing

**Figure 2:**
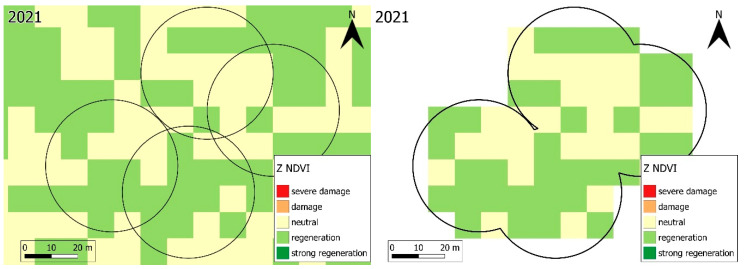
Study areas made from the ICP Forests monitoring plots. The four satellites of the main plot were merged into one to be used as clipping rasters.

In order to compare years with and without forest damage, the vegetation periods of the years 2017 to 2023 were analysed at the national and landscape level using high-resolution (10 × 10 m) Sentinel-2 satellite images. Forest health status estimation expressed as Z NDVI vegetation index calculated from the satellite imagery was performed for the study areas and period in the Google Earth Engine cloud platform (Figure 2).

Among the geodatabases available in the Google Earth Engine cloud repository [22], space images from the European Space Agency (ESA) Sentinel-2 satellites [5] were used to determine forest health and its changes. Our approach uses multispectral satellite imagery from the high-resolution Sentinel-2A, B, and C MSI sensors, which are accessed, stored, processed, analysed, and displayed in the cloud. The Sentinel-2 bands we applied in this study have 10 × 10 m resolution: RED (band 4) and Near-Infrared (NIR) (band 8). Other bands have 20 × 20 m resolution, like SWIR or Vegetation Red Edge, while for aerosol and water vapour detection, 60 × 60 m bands are used.

Cloud masking involved multiple steps to ensure high-quality, cloud-free imagery. Firstly, we applied prefiltering CLOUDY_PIXEL_PERCENTAGE metadata, filtering out images with more than 5% cloud cover. Secondly, we used the quality bitmask QA60 targeting bits 9, 10, and 11, which correspond to clouds and cirrus features. Thirdly, we utilised the Scene Classification Layer (SCL) band to exclude cloud shadows. In SCL, we applied bits 3, 7, 8, 9 to exclude cloudy and shady pixels.

The compositing period is the vegetation season from April to October, and a median reductor was applied to create a single, cloud-free image for each year.

To estimate photosynthetic activity, we used the Normalised Vegetation Index (NDVI) (1), calculated according to [23]:

(1) $$NDVI=\frac{NIR-RED}{NIR+RED}$$

where NIR is the near-infrared and RED is the surface reflectance in the red coupling channel. An NDVI scale of 0 to 1 is used to indicate forest health, where low values indicate vegetation with no vegetation on the ground surface or vegetation with weak photosynthetic activity, and higher values indicate healthy forest vegetation with strong photosynthesis.

NDVI values are quite variable over time and represent the state of a moment in time, rather than a deviation from the average. For the latter, we use the standardised version of NDVI (Z NDVI) (2), calculated using the following formula [24]:

(2) $$NDVIZ=\frac{NDVI-{NDVI}_{mean}}{{NDVI}_{std}}$$

where NDVI is the median composite for the growing season of the year, NDVI_mean_ is the mean for the period 2017–2023, and *NDVI*_std_ is the standard deviation for the same period. Negative values of Z NDVI indicate degradation, and positive values indicate recovery.

By classifying the Z NDVI values, the distribution of pixels belonging to a given class provides an indication of the overall forest condition on a spatial statistical basis. Five classes were made:

Z NDVI < −2: severe forest damage;
Z NDVI < −1: forest damage;
Z NDVI < 0: unchanged, neural condition;
Z NDVI < 1: improvement, regeneration;
Z NDVI > 1: strong regeneration.

### 2.5. Climate Data

**Figure 3:**
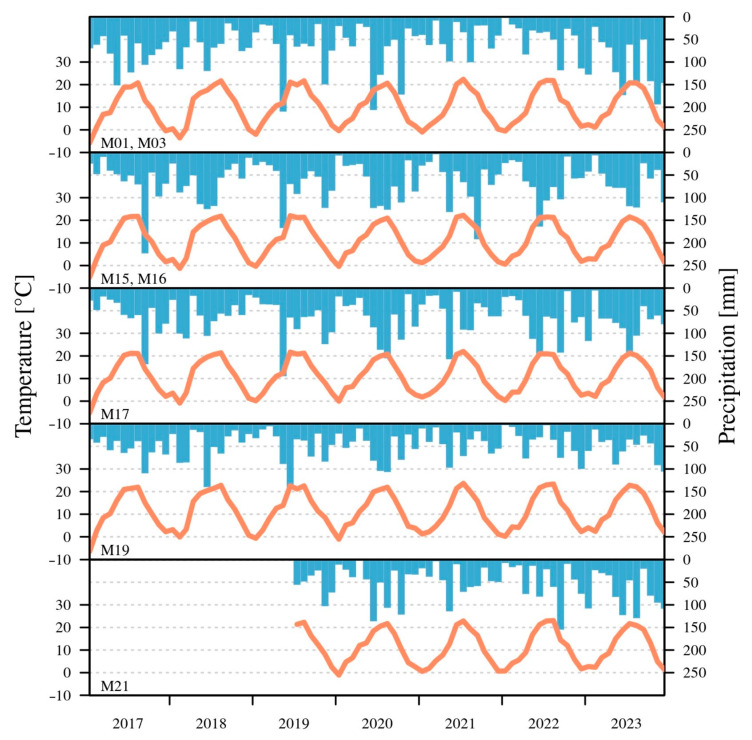
Yearly regime of temperature and precipitation in Level II plots between 2017 and 2023. The orange line stands for temperature, while the blue bar does for precipitation.

Meteorological records from Level II open-field plots (Figure 3) revealed a consistent increase in mean annual temperature between 2017 and 2023. In 2022, thermal extremes were pronounced due to a long drought, with 62 heat days (Tmax ≥ 30 °C) and 14 hot days (Tmax ≥ 35 °C) on the Great Hungarian Plain. Monthly means in 2022 exceeded the 1991–2020 climatological baseline, and annual means were above the 2017–2021 average across all sites. A warm, precipitation-deficient period occurred from January to April 2022. The highest site-level temperatures were generally recorded at the Black locust stand (M19). Drought events were detected in May–July of both 2017 and 2022 at the Common beech (M01), Sessile oak (M03), and Black locust (M19) plots, while the Turkey oak site (M21) experienced drought in April, July, and October 2022. The Black locust plot (M19) was subject to prolonged and severe drought. In 2023, the interannual temperature range was lower at all plots compared to 2022, with a 0.3 °C decrease in mean temperature at the Black locust plot (M19) in the central region [25,26].

Regarding annual precipitation in 2022, it was 8% below the 2017–2021 mean across all plots, with particularly low values in central Hungary [26,27]. The minimum total (471.5 mm) was measured at the Black locust stand (M19), whereas maxima were recorded in southwestern Hungary at the Common beech site (M17) in both 2022 and 2023. Precipitation totals in 2023 exceeded 2022 by 30%. Western plots, including Scots pine (M15), Sessile oak (M16), and Common beech (M17), showed reduced drought sensitivity in 2022 [28,29].

The interception computed by the throughfall was measured by 12–16 funnels, which were set up in a regular system, and by five buckets in Level II plots. The annual interception value in drought years decreased significantly compared to the average of 2017–2023. There was significant leaf loss due to prolonged drought in 2022. The interception rate decreased due to the early summery leaf loss by 5% in sample areas. In 2023, the yearly interception was higher by 5–8% in Level II monitoring plots due to wetter weather.

### 2.6. Validation

To evaluate how well remote-sensing-based indicators capture actual crown condition at the plot scale, correlate the mean NDVI values (or their anomalies) with the observed defoliation percentages recorded in the field [8]. Where defoliation is classified categorically (e.g., damaged vs. healthy), calculate classification accuracy statistics using confusion matrices with field assessments as reference data. When using continuous defoliation values, apply regression-based performance metrics [30]. Examine any strong discrepancies between satellite- and field-based signals: some remote-sensing false positives arise from logging events or vigorous understory vegetation, while field plots could miss subtle early discolouration detectable from above. Thresholds for damage detection may need adjusting or supporting with auxiliary information (e.g., excluding thinned and clearcut areas based on GIS layers) [31].

Comparing the remote-sensing-derived metrics with field data at the plot level, correlate average NDVI or anomaly scores with the recorded defoliation % [8]. Calculate accuracy metrics if classifying damage (confusion matrices using field observations as truth). For continuous defoliation estimates, use regression metrics [30]. Identify outliers or mismatches and investigate causes; a false alarm might be due to a harvest operation or understory growth; conversely, field surveys might miss early subtle discolouration that satellites pick up. Refine the index thresholds or add ancillary data if needed (such as excluding recently thinned stands using forest management GIS data) [31].

### 2.7. Statistical Analysis

A confusion matrix is a table used to evaluate the performance of a classification model. It compares the model’s predicted classifications against the actual labels and shows how many predictions fall into each category. From this matrix, you can calculate performance metrics like total accuracy, precision, sensitivity, specificity, and F1 score [32].

A True Positive (TP) is an outcome where the model correctly predicts the positive class. In other words, the case is positive and is correctly identified as a position. A True Negative (TN) is an outcome where the model correctly predicts the negative class. A False Positive (FP) occurs when the model incorrectly predicts the positive class for a negative instance. A False Negative (FN) occurs when the model fails to predict the positive class for an actual positive instance.

Precision (3) is the proportion of correctly predicted positive cases among all positive predictions:

(3) $$Precision=\frac{TP}{TP+FP}$$

Sensitivity (or Recall, 4) is the proportion of actual positive cases correctly identified by the model:

(4) $$Sensitivity=\frac{TP}{TP+FN}$$

Specificity (5) stands for the proportion of actual negative cases correctly identified as negative:

(5) $$Specificity=\frac{TN}{TN+FP}$$

Total Accuracy (6) is the proportion of all correct predictions (both positive and negative) out of all predictions made:

(6) $$\mathrm{Total}\;\mathrm{Accuracy}=\frac{TP+TN}{TP+TN+FP+FN}$$

The F1 Score (7) is the harmonic mean of precision and recall. It provides a single combined metric of a classifier’s performance by balancing the precision and the recall.

(7) $$F1=\frac{2TP}{2TP+FP+FN}$$

R-squared (R^2^), or the coefficient of determination (8), is a regression metric that represents the proportion of variance in the dependent variable that is explained by the independent variable in the model. An R^2^ of 1 indicates the model perfectly explains all variability of the outcome, whereas 0 indicates the model explains nothing.

(8) $$R^{2}=1-\frac{RSS}{TSS}$$

where RSS is the residual sum of squares and TSS is the total sum of squares of the outcome about its mean.

Root Mean Squared Error (RMSE) is a regression metric that measures the average magnitude of the prediction error in the same units as the target variable, as the standard deviation of the residuals (9). RMSE is the square root of the average of the squared differences between predicted and actual values. A lower RMSE indicates a better fit.

(9) $$RMSE=\sqrt{\bar{{\left(f-o\right)}^{2}}}$$

where f is the forecasts (expected values or unknown results) and o is the observed values (known results).

Cohen’s Kappa (10) is a statistic that measures inter-rater agreement for categorical items, adjusting for agreement that could happen by chance. It is considered more robust than a simple per cent agreement because it considers the probability of random agreement between raters. Cohen’s Kappa is used when two raters (or two methods) classify items, and it answers how much better the agreement is than what you’d expect by chance. A k value of 1 indicates perfect agreement, and 0 indicates no agreement.

(10) $$k=\left(p_{o}-p_{e}\right)∕\left(1-p_{e}\right)$$

where p_o_ is the relative observed agreement among raters and p_e_ is the hypothetical probability of chance agreement.

## 3. Results

The results are based on data from national forest inventories, ICP Forests Level II (intensive) and Level I (extensive) plots. Satellite imagery was used to scale and validate ground-based observations. Combined, these sources revealed spatial patterns and trends in forest health, including drought-related stress and increased defoliation.

### 3.1. Satellite Imagery

**Figure 4:**
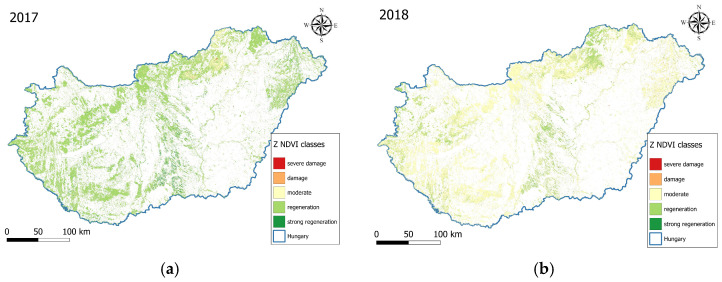

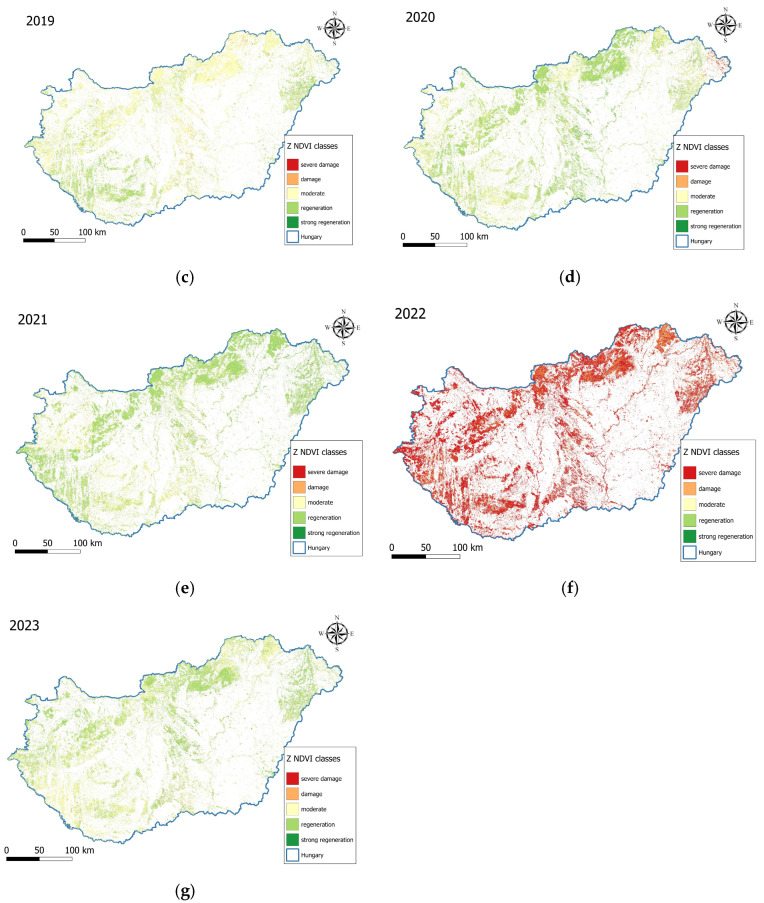
Z NDVI maps for the forest area in Hungary, 2017–2023 (**a**–**g**). It can be seen that, following the generally good condition of the forests in 2017 and 2021, the significant negative impact of the droughts in 2018 and 2022 is observable, as well as the regeneration in the wetter years of 2019 and 2023.

Thanks to the 10 × 10 m spatial resolution of Sentinel-2, we could observe the changes within the forest patches in the landscape-level studies (Figure 4). The Z NDVI values of the vegetation period composites differ significantly due to the drought in 2022. Depending on the extent of potential forest damage, more severe forest patches are marked in orange and red, while less or no damage is marked in lemon yellow and healthy patches in green.

Comparing the classes, one can see the very significant negative effects of the 2018 and 2022 droughts, after the generally good condition of the forests in 2017 (95% good condition), where we first recorded a 70% negative change to the medium class, and in 2022 67% to the significant deterioration class. The rainier year of 2023 also saw a regeneration (+50% moved to the medium class). The difference between 2021 and 2022 is most significant, where the severe damage class replaced the good health class of 70% with the same proportion of 67%. The impact of drought is therefore evident, as is the improvement in 2023 due to more rainfall, where half of the pixels moved to the medium health class.

**Figure 5:**
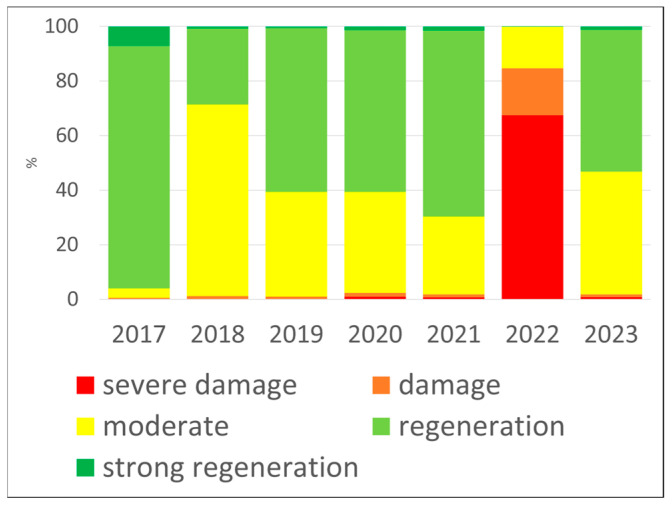
Cumulative bar charts of NDVI classes for the forest area in Hungary, 2017–2023. It can be seen that after the generally good condition of the forests in 2017, 2019, 2020, and 2021, the significant negative impact of the drought in 2018 and 2022 can be observed, as well as the regeneration in the wetter years of 2019 and 2023.

Between 2017 and 2023, the distribution of Z NDVI classes in Hungarian forests showed interannual variability (Table 2). In 2017, the forest health state was overwhelmingly favourable, with 96% of pixels falling in the positive categories. Controversially, 2018 was a drier year, where 70.3% of the forest in a moderate state. Later in 2019, 2020, and 2021, a positive trend was observed, while this pattern reversed in 2022. In that year, the extremely severe drought conditions left roughly 84.7% of pixels in the negative range, of which 67.5% was severe damage, highlighting the severity of the event. By 2023, the vegetation response improved: 45% of the pixels shifted back into the moderate range, while the remainder still reflected stress through negative values. The regeneration of the forest happened due to wet conditions, as the precipitation totals equalled or exceeded the previous year’s annual sum and the long-term mean as well. These rains supported forest recovery significantly, as captured also by the 2023 Z NDVI distribution with 53.2% regeneration and strong regeneration class pixels. The analysis of percentile-class shifts indicates a deterioration in 2018 and 2022, succeeded by a recovery in 2019 and 2023 (Figure 5). These results clearly demonstrate the impact of drought, as well as the forest health improvement in 2023, attributable to increased precipitation.

### 3.2. ICP Forests Level II

**Figure 6:**
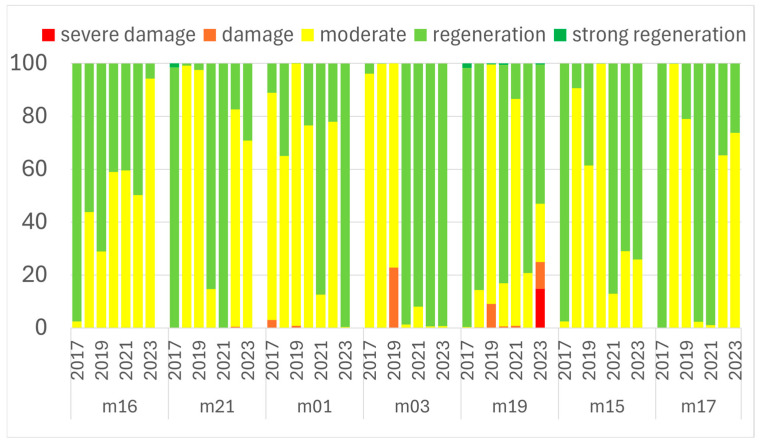
Z NDVI classes of ICP level II plots in Hungary for 2017–2023.

Analysing seven Level II forest monitoring plots revealed varying health states, with Bajánsenye (M16), Gyöngyössolymos (M03), and Szentpéterfölde (M17) initially showing the best vitality based on vegetation index values (photosynthetic activity), while Kecskemét (M19), Biatorbágy (M21), and M16 showed deterioration. The severe 2022 drought visibly impacted M19 and M16 in 2023, and M17 across both 2022 and 2023, although M03 improved significantly and remained unaffected by drought. Field-based ICP defoliation reports showed the excellent, drought-unaffected state of M21 and M03; however, they showed M19 in constant decline, peaking at 62% defoliation in 2022, and documented a volatile history for M17, which suffered damage in 2017 (late frost or wind) and again in 2021 (wind) and 2022 (drought, 24% defoliation). Plot M16 displayed complex changes that sometimes contrasted with satellite data (e.g., field-reported regeneration in 2022, while satellite noted a decline in 2023), but overall, a good general agreement was found between the field and satellite data, though accuracy could be improved (Figure 6).

At plot M16, from 2015 to 2017, there was a gradual deterioration in average defoliation from year to year. From 2018 onwards, this trend was reversed, with a decrease in defoliation compared to previous years. The improving trend continued in 2019 and 2020, but in 2021, a slightly higher average foliage loss was recorded due to unfavourable weather conditions. In 2022, however, the proportion of healthy foliage increased again, and the average defoliation decreased.

In 2017, at plot M01, we again recorded a higher rate of defoliation and an outstanding mortality. Subsequently, we measured data indicating improved health from 2018 to 2020. In 2021, however, significant leaf loss was again observed, mainly due to abiotic causes. In 2022, despite the drought, the average leaf loss and branch mortality decreased. From 2018 to 2020, the data showed an annual improvement at M03, but in 2021, the percentage of fully healthy canopies decreased slightly, although still with very favourable values. In 2022, the proportion of asymptomatic canopy decreased further to 78.4%.

At plot M19, the average defoliation rate increased to 43.1% in 2021. In 2022, the situation deteriorated further, and the average defoliation rate reached 62%. The branch dieback rate also showed a steadily increasing trend, reaching 28.8% in 2021 and 33.6% in 2022, in line with the increase in defoliation. These values indicate poor stand health.

From 2014 onwards, there was a gradual improvement in the stand at plot M15. Between 2018 and 2022, the overall condition of the stand shows a balanced picture of good health, both in terms of defoliation categories and average defoliation.

**Figure 7:**
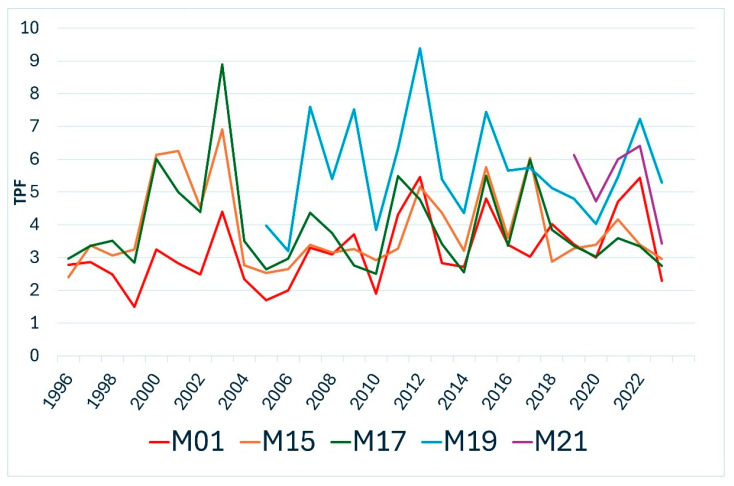
TPF graphs for regions of Hungary with ICP Level II plots.

Year 2017 was an exceptionally unfavourable year for beech in plot M17, as shown by the foliage loss data, due to stormy weather and late frosts. From 2018 onwards, a steady improvement in the pseudo-status of the stand in terms of health is observed. In 2021, a slight deterioration was observed, clearly due to windy weather. In 2022, although not indicated by the TPF value, an average leaf loss of 24.1% was recorded, clearly due to dry, droughty weather. Based on the time trend of the data, activity was most positive in 2017 and 2021, while from 2022 onwards, significant negative changes were observed (Figure 7).

### 3.3. ICP Forests Level I

We examined our study with the 78 ICP Forests Level I plots in Hungary. The data of Level I plots can be analysed according to different criteria. One important approach is to examine changes over time, including trends, outliers, and groupings within the dataset. By breaking the data into different years and observing variations, we can identify significant patterns that highlight shifts in activity levels (Table 3).

Seeing the trends over time, the data in 2017 shows mostly positive values, indicating that the initial state of the forests was ideal. However, in 2018 and 2019, a decline was observed, with several rows showing a value of 0, which may suggest a decrease in activity. In 2020 and 2021, the data became more variable, but many rows show regeneration, suggesting a return of recurrent activity. A major shift occurs in 2022 and 2023, where most rows display negative values (severe damage and damage), clearly indicating a downturn in data points and a deterioration in forest conditions.

Examining our data, positive forest health classes appear more frequently in 2017 and 2021, signifying years of increased positive outcomes or activity. In contrast, negative values referring to damage started appearing regularly from 2022 onwards and became dominant, indicating a clear negative shift in the dataset.

Another factor to consider is the missing values in some rows, particularly for plots 652, 941, 961, 969, 985, and 994. Missing data might be due to errors in data collection, incomplete records, or other external factors that need further investigation.

Comparing the data year by year reveals clear differences in trends. In 2017, most rows contain positive values, indicating prominent levels of positive activity. The years 2018 and 2019 introduce more variation, with an increase in neutral values, suggesting a more balanced situation. A resurgence in positive activity is observed in 2020 and 2021, as these years contain more positive classes. However, 2022 marks a significant turning point, with almost all rows showing negative values, suggesting a drastic decline. By 2023, some signs of regeneration appear, though the overall trend remains uncertain.

Some data series display consistently higher numbers of positive values, indicating better performance or more favourable outcomes over the years. For instance, plot 3 shows positive values in 2017 and 2021, with only a minor negative dip in 2022 before recovering in 2023. Similarly, plot 21 maintains a strong positive trend from 2017 to 2021 before experiencing a negative shift in 2022, but rebounds in 2023. Other plots with similar patterns include 219, 328, and 332, all of which show positive trends up to 2021, a decline in 2022, and some level of recovery in 2023.

Conversely, some data series exhibit more negative values, indicating declining performance. For example, plot 170 has only two positive years (2019 and 2021) but experiences a sharp negative turn in 2022 and 2023. Plot 281 sees a strong positive year in 2019, but later declines significantly. Similarly, plot 122 starts with a positive trend but drops sharply in 2022. Other plots, such as 260 and 781, follow a similar trajectory, with early positive trends followed by a marked decline in 2022.

Overall, the data highlights a significant shift in trends, with early years showing positive activity, followed by fluctuating trends, and a major downturn in 2022. The presence of missing data, outliers, and significant variations across different plots provides further depth to the analysis. Future studies should focus on understanding the causes behind these shifts and identifying potential strategies for recovery.

### 3.4. Statistical Analysis Results

We analysed statistical parameters of our dataset to show the strength of the connection between remotely sensed and ground-based data. In Table 4 and Table 5, all elements of the confusion matrix, F1-score, Cohen’s Kappa, R^2^ and RMSE mean values are presented for Z NDVI and ICP Forests Level I and II defoliation, respectively. Pooled values were also calculated for every year and plot (Table 6 and Appendix A Table A1), resulting in a theoretical 7 × 7 (Level II) and 78 × 7 (Level I) plot values. In practice, 45 values were missing from Level I and 2 from Level II.

We found strong agreement among the study areas. Plot M19 demonstrated excellent and stable categorical agreement, with its kappa value consistently ranging between 0.70–0.78 over the seven years, achieving a 94.2% F1-Score. This high kappa strongly validates that the remote sensing NDVI and the field defoliation are reliably interchangeable for establishing the same damage classification in this area. The method was consistently effective in detecting severe damage and damage with high Precision and Specificity. While there was moderate and varying accuracy at plots M16, M01, and M03. Plot M16 achieved the highest kappa, 0.52, while plots M01 and M03 showed average kappa values below 0.2. The reliability of damage detection (with F1-Scores up to 71%) showed uncertainty, thus further optimisation is needed. The worst performance was experienced on plots M15, M17, and M21, which yielded the weakest results. The average kappa remained below 0.1, indicating weak agreement with low F1 values and a high rate of FN.

Our model has an average Total Accuracy value of around 61%, and an R^2^ value ranging between 0.34–0.38 (Table 7). Although these results indicate that Sentinel-2 data have predictive power regarding defoliation, our performance is lower than the results published in the literature. The referenced Polish and Slovakian studies [3,33], which also used ICP Forests and Sentinel-2 data, achieved better classification accuracy, with a Total Accuracy between 75–78%. Furthermore, their regression models showed stronger explanatory power, with R^2^ values in the range of 0.53. This difference suggests that their methods (machine learning) and a broader set of spectral indices were more effective at capturing the relationship between satellite data and defoliation.

We calculated linear regression with R^2^ and RMSE values to highlight how TPF explained and predicted the variation in the Z NDVI (Table 7). Among the studied sites, M21 showed the strongest relationship (R^2^ = 0.5, RMSE = 6.87%), indicating that more than half of the Z NDVI variation is driven by climatic factors, and the model provides highly accurate predictions. Plots M19 and M15 (R^2^ = 0.34–0.28) showed moderate explanatory power, while M01 and M17 performed poorly, suggesting that complex influences dominate NDVI dynamics at these locations.

## 4. Discussion

The integration of Sentinel-2 satellite imagery with ICP Forests Level I and II monitoring systems marks a significant advancement in Europe’s approach to forest health assessment. The high resolution of Sentinel-2 and its frequent image acquisition enable the detection of defoliation, drought stress, and gradual forest decline over wide areas—capabilities that complement the spatial limitations of traditional ground-based surveys [2,34].

Future research is likely to focus on harmonising satellite-derived indices, such as NDVI and NDMI, with visual defoliation scores and tree physiological data collected by ICP Forests. Studies have shown promising results linking Sentinel-2 metrics with crown condition trends, particularly for species like European beech [35,36].

Recent advancements in remote sensing and Machine Learning have further improved the detection and monitoring of land use, forest cover, and forest disturbances [37]. Transformer-based models trained on extensive Sentinel-2 time series data have demonstrated high accuracy in identifying forest disturbances in Central Europe [38].

It is recommended to utilise both greenness and moisture indices together, as each can fail in certain cases (NDVI saturates in dense canopies; NDMI can be insensitive in non-drought stress) [14]. Filtering out cloud/atmospheric effects is critical. the utilisation of Level 1C products is preferable to avoid potential over-correction artefacts in forested areas [3]. Multi-temporal compositing (median of several clear images) is preferred over single-date images to mitigate cloud gaps and ephemeral effects [39]. For anomaly detection, define the reference normal period carefully (ideally using multiple years of data to capture natural variability) and focus on the same phenophase when comparing indices. Incorporating species-specific knowledge can also improve results: e.g., calibrate index thresholds separately for conifers vs. broadleaves, if possible, since their reflectance and defoliation responses differ [8]. Finally, close collaboration with forest health experts (as emphasised by ICP Forests) is key; the algorithms should be tuned to detect changes relevant to forest managers and conform to standards (like damage classes) [9]. This ensures that remote sensing products (maps of disturbance or defoliation) are interpretable in the same framework as traditional surveys, facilitating adoption in operational monitoring.

Recent studies conclusively show that Sentinel 2 has become an invaluable tool for forest health monitoring in synergy with the ICP Forests program. By extracting vegetation indices (NDVI, NDMI, red-edge indices) and analysing their time-series behaviour, researchers can detect forest condition changes such as drought stress, pest-induced defoliation, and storm damage, often as they happen or shortly after. These satellite-derived insights, when validated against ICP Level I & II observations, allow for scaling up point-based assessments to continuous maps, improving our spatial understanding of forest disturbances [30].

Despite the ongoing popularity of the NDVI as a greenness indicator, emerging indices like NDMI and red-edge-based vegetation indices are set to improve future remote sensing of vegetation by providing complementary data. NDMI uses near-infrared and shortwave infrared reflectance to measure vegetation water content, assisting in drought monitoring and revealing moisture dynamics that NDVI alone cannot capture [40]. Meanwhile, red-edge spectral indices have demonstrated higher sensitivity to plant physiological conditions and a reduced tendency to saturate in dense vegetation, compared to NDVI [41].

Best-practice methodologies involve careful preprocessing, index combination, anomaly detection algorithms (NDVI change or Z NDVI maps), and iterative calibration with ground data to reach accuracies on the order of the field measurements. In practical terms, a Sentinel 2-based monitoring pipeline can serve as an early warning system for drought effects or insect outbreaks, and as a cost-effective means to complement annual ICP surveys with more frequent information. Integrating such remote sensing approaches into the ICP Forests process and national forest health reports is recommended by experts. This integrated monitoring will enhance our ability to respond to forest disturbances under changing climate conditions, supporting more initiative-taking forest management and conservation strategies.

Beyond drought stress detection, Sentinel-2 is increasingly used for identifying biotic disturbances such as bark beetle infestations. The red-edge and shortwave infrared bands have proven especially useful for detecting early-stage insect damage before visible browning occurs [42]. The integration of in situ data with Sentinel-2 imagery has also proven effective in assessing soil quality and detecting post-fire soil degradation [43]. Furthermore, the combination of Sentinel-2 data with airborne laser scanning has improved the mapping of old-growth forests, providing essential information for conservation efforts [44]. Combining satellite data with ecological models can also help predict outbreak areas, supporting early warning systems.

Looking ahead, multi-sensor integration and upcoming radar and hyperspectral missions (Biomass, FLEX) will enhance forest health monitoring. These innovations, combined with robust ground validation from ICP Forests, will allow for more precise, timely, and scalable forest condition monitoring across Europe [34]. Remote sensing technologies offer the potential for more timely and accurate assessments of forest health, enabling more effective forest management strategies.

## 5. Conclusions

Based on the results, the satellite remote sensing methodology for drought assessment has been successfully applied for the period 2017–2023. The NDVI Z was suitable for detecting changes because the 2018 and 2022 droughts were visible in both the spatial images and the statistics, as well as Level I and Level II plots.

Overall, results show that Sentinel-2 satellite data have a generally reliable but spatially variable relationship with ground-based observations. We found high categorical agreement and accuracy for certain sites (0.78 kappa or 0.94 F1), which supported the efficiency of the remote-sensing approach for defoliation detection. However, other plots showed weaker performance, represented by lower kappa values and reduced reliability of the method. The overall model accuracy of 61% points to the fact that Sentinel-2 NDVI captures part of the variability in forest defoliation but cannot explain every aspect of the forest health change in recent form.

During the data processing, we faced certain limitations of the method, which partly explain the abovementioned results. Cloud cover introduces inconsistencies between years, and NDVI-based indices cannot fully distinguish between overlapping stressors such as drought, wind, and biotic disturbances. The spatial resolution of Sentinel-2 also restricts single tree-level analysis, while ICP Level II is primarily made on that level. On the other hand, national damage inventories also proved to be useful for forest damage detection [33,45].

A new Remote Sensing Working Group was created in 2025 within the ICP Forests program to support traditional ground-based forest monitoring data with remote sensing technologies, including satellites, drones and AI. The new group aims to enhance the effectiveness and precision of forest condition monitoring throughout Europe, particularly in relation to the impacts of climate change, forest decline, and air pollution. This group aims to provide a novel methodological approach to integrating remote sensing data into the ICP forest monitoring. This is an important step forward for the ICP Forests program, as it enables the first large-scale and continuous monitoring of forest ecosystems [46].

Future work should improve cloud masking, harmonise satellite indices with forest health data on the single tree level, and integrate multi-sensor approaches, including radar and lidar observations, to make denser time series. Advances in Machine Learning and Deep Learning could further support near-real-time monitoring, offering more precise and flexible guidance for forest management and policies.

A Random Forest-based study evaluated the combined effectiveness of Landsat, Sentinel-2, and Sentinel-1 time series data for detecting bark beetle infestations, comparing performance across combinations. The research found that Sentinel-2 data alone offered the best spatial accuracy, achieving 93% TA. The Random Forest classifier provided timely detection of the infestation at the onset of the outbreak [37].

A Central European study employs the Transformer Deep Learning (DL) architecture on Sentinel-2 time series data for the effective and accurate detection of forest disturbances, such as bark beetle infestations, in Central Europe, achieving an F1-score 0.72 and detecting small-sized damage. The DL model confirms the efficacy of Transformers for fine-grained forest monitoring [47].

Our results so far showed that large-scale changes in forest health can be monitored using appropriately designed and interpreted remote sensing methods. At the same time, it has also been confirmed that these remote sensing methods only provide a more accurate picture of the health status of forests and their spatial changes when combined with and supplemented by field observations. Ground observations are essential for the precise identification of different types of forest damage and for determining their spatial extent and intensity. Remote sensing data, on the other hand, provides faster data covering a larger area. With the continuous development of the method and its integration into practice, we can expect better results in the future.

As for the climate of Europe, according to the Copernicus programme, the warming process has been occurring at a nearly two times greater rate than the global average rate, resulting in extreme heatwaves, wildfires, floods, and droughts. Warming is going to accelerate further according to predictions, leading to even more severe and frequent extremes. Central Europe may experience summers that are drier but storms that are heavier. Although there are clear warnings, adaptation takes place [48].

## Figures and Tables

**Table 1:** Defoliation categories of ICP Forests.

Defoliation Category	Defoliation %
None	0–10
Weak	>11–25
Medium	>26–60
Severe	>61–99
Dead tree	100

**Table 2:** Distribution of Z NDVI classes (%) across forest areas in Hungary, 2017–2023.

Z NDVI Class	Forest Health	2017	2018	2019	2020	2021	2022	2023
<−2	severe damage	0.1	0.2	0.1	1	0.8	67.5	0.8
−1	damage	0.4	1	0.9	1.4	1	17.2	1
0	moderate	3.4	70.3	38.4	36.9	28.5	15.2	45
1	regeneration	88.8	27.8	60.1	59.2	68.2	0.1	52
2<	strong regeneration	7.2	0.8	0.6	1.4	1.5	0	1.2

**Table 3:** Z NDVI classes for ICP level I plots in pieces of compartments.

Class	2017	2018	2019	2020	2021	2022	2023
severe damage						66	
damage		1				7	1
neutral status	4	57	51	33	21		38
regeneration	67	15	21	39	51		33
strong regeneration	3		1	1	1		1

**Table 4:** Confusion matrix of Z NDVI and ICP Forests level I defoliation data.

Year	Kappa	TP	FP	FN	TN	Precision	Sensitivity	Specificity	Total Accuracy	F1	R^2^	RMSE
2017	0.32	0.51	0.49	0.49	0.68	0.51	0.51	0.58	0.63	0.51	0.41	21
2018	0.37	0.50	0.50	0.50	0.64	0.50	0.50	0.57	0.60	0.50	0.46	20
2019	0.27	0.38	0.62	0.62	0.67	0.38	0.38	0.54	0.60	0.38	0.31	21
2020	0.31	0.42	0.58	0.58	0.66	0.42	0.42	0.57	0.61	0.42	0.37	18
2021	0.36	0.47	0.53	0.53	0.70	0.47	0.47	0.60	0.63	0.47	0.42	19
2022	0.29	0.40	0.60	0.60	0.66	0.40	0.40	0.54	0.59	0.40	0.35	20
2023	0.31	0.42	0.58	0.58	0.64	0.42	0.42	0.56	0.60	0.42	0.35	20

**Table 5:** Confusion matrix of Z NDVI and ICP Forests level II defoliation data.

Year	Kappa	TP	FP	FN	TN	Precision	Sensitivity	Specificity	Total Accuracy	F1	R^2^	RMSE
2017	0.32	0.51	0.49	0.49	0.68	0.51	0.51	0.58	0.63	0.51	0.4	21
2018	0.37	0.50	0.50	0.50	0.64	0.50	0.50	0.57	0.60	0.50	0.5	20
2019	0.27	0.38	0.62	0.62	0.67	0.38	0.38	0.54	0.60	0.38	0.3	21
2020	0.31	0.42	0.58	0.58	0.66	0.42	0.42	0.57	0.61	0.42	0.4	18
2021	0.36	0.47	0.53	0.53	0.70	0.47	0.47	0.60	0.63	0.47	0.4	19
2022	0.29	0.40	0.60	0.60	0.66	0.40	0.40	0.54	0.59	0.40	0.4	2
2023	0.31	0.42	0.58	0.58	0.64	0.42	0.42	0.56	0.60	0.42	0.4	2

**Table 6:** Comparison of ICP Forests level I and II confusion matrices.

Level	Kappa	TP	FP	FN	TN	Precision	Sensitivity	Specificity	Total Accuracy	F1	R^2^	RMSE
Level I	0.32	0.44	0.56	0.56	0.65	0.44	0.44	0.57	0.61	0.44	0.38	20
Level II	0.25	0.38	0.62	0.62	0.65	0.38	0.38	0.56	0.60	0.38	0.34	23

**Table 7:** Regression analyses of TPF and Z NDVI classes.

Plot	R^2^	RMSE
M01	0.01	12.59
M15	0.28	10.22
M17	0.02	13.31
M19	0.34	10.56
M21	0.50	6.87

## Data Availability

The original contributions presented in this study are included in the article. Further inquiries can be directed to the corresponding author.

## Abbreviations

The following abbreviations are used in this manuscript:

AI	Artificial Intelligence
DL	Deep Learning
ESA	European Space Agency
FAPAR	Fraction of absorbed photosynthetically active radiation
FN	False Negative
FP	False Positive
ICP Forests	International Co-operative Programme on Assessment and Monitoring of Air Pollution Effects on Forests
LAI	Leaf Area Index
NASA	National Aeronautics and Space Administration
NDMI	Normalised Difference Moisture Index
NDVI	Normalised Difference Vegetation Index
RMSE	Root Mean Square Error
RF	Random Forest
RSS	Residual Sum of Squares
TN	True Negative
TP	True Positive
TSS	Total Sum of Squares
Z NDVI	Standardised Difference Vegetation Index

## Appendix A

**Table A1:** Confusion matrix of ICP Level II plots for each year.

Plot	Year	Kappa	TP	FP	FN	TN	Precision	Sensitivity	Specificity	Total Accuracy	F1	R^2^	RMSE
M19	2017	0.75	91.5	8.5	8.5	83.1	91.5	91.5	90.7	86.2	91.5	0.92	0.08
	2018	0.7	88	12	12	79.9	88	88	87	83.1	88	0.88	0.09
	2019	0.73	90.1	9.9	9.9	81	90.1	90.1	89.1	85	90.1	0.9	0.07
	2020	0.78	94.2	5.8	5.8	83.3	94.2	94.2	94.4	87.5	94.2	0.93	0.06
	2021	0.76	92	8	8	82.5	92	92	91.1	86.8	92	0.91	0.08
	2022	0.71	89.5	10.5	10.5	80.3	89.5	89.5	88.4	83.9	89.5	0.89	0.09
	2023	0.77	93.1	6.9	6.9	83.7	93.1	93.1	92.3	87.1	93.1	0.9	0.07
M16	2017	0.19	35.1	64.9	64.9	70.1	35.1	35.1	51.9	63.4	35.1	0.25	0.25
	2018	0.38	51	49	49	75.1	51	51	60.6	69.2	51	0.48	0.18
	2019	0.5	68.3	31.7	31.7	84.9	68.3	68.3	72.8	75.8	68.3	0.65	0.15
	2020	0.4	54.7	45.3	45.3	76	54.7	54.7	62.7	70.1	54.7	0.55	0.17
	2021	0.52	71.4	28.6	28.6	86.1	71.4	71.4	75.1	77	71.4	0.69	0.14
	2022	0.39	53.9	46.1	46.1	75.6	53.9	53.9	61.9	69.8	53.9	0.6	0.16
	2023	0.12	29.8	70.2	70.2	67	29.8	29.8	48.8	59.2	29.8	0.4	0.21
M15	2017	0.45	60.5	39.5	39.5	78.9	60.5	60.5	66.6	72.8	60.5	0.55	0.15
	2018	0.05	15.2	84.8	84.8	58	15.2	15.2	38.6	55	15.2	0.15	0.25
	2019	−0.08	9.1	90.9	90.9	51.2	9.1	9.1	35.9	49.3	9.1	0.05	0.32
	2020	−0.15	4.5	95.5	95.5	47.3	4.5	4.5	33.1	45.1	4.5	0.02	0.38
	2021	0.25	41.2	58.8	58.8	71.7	41.2	41.2	55	65	41.2	0.3	0.22
	2022	0.04	12	88	88	57.1	12	12	39.3	54.1	12	0.12	0.3
	2023	0.02	10.8	89.2	89.2	55.9	10.8	10.8	38.5	52.9	10.8	0.09	0.35
M17	2017	0.01	11	89	89	53.3	11	11	37.4	51.2	11	0.1	0.3
	2018	0.05	16.5	83.5	83.5	58.2	16.5	16.5	39.6	55.4	16.5	0.15	0.28
	2019	−0.09	8.8	91.2	91.2	50.8	8.8	8.8	35.7	48.9	8.8	0.02	0.35
	2020	0.31	46.2	53.8	53.8	73.5	46.2	46.2	57.7	68.1	46.2	0.45	0.18
	2021	0.18	34	66	66	69.3	34	34	49.8	62.5	34	0.3	0.25
	2022	0.08	20.1	79.9	79.9	60.1	20.1	20.1	42.9	56.7	20.1	0.2	0.3
	2023	0.07	18.5	81.5	81.5	59.3	18.5	18.5	41.6	55.8	18.5	0.15	0.32
M01	2017	0.2	36.5	63.5	63.5	69.1	36.5	36.5	51.3	63.8	36.5	0.3	0.22
	2018	0.23	39.2	60.8	60.8	70.6	39.2	39.2	53.7	65.5	39.2	0.35	0.18
	2019	0.02	13.1	86.9	86.9	55.4	13.1	13.1	37.3	52.1	13.1	0.1	0.25
	2020	0.04	15	85	85	56.5	15	15	38.8	54	15	0.15	0.24
	2021	0.1	24.5	75.5	75.5	62	24.5	24.5	44.9	58	24.5	0.2	0.23
	2022	0.15	30.1	69.9	69.9	66.8	30.1	30.1	50.6	60.5	30.1	0.25	0.2
	2023	0.13	27.8	72.2	72.2	65.5	27.8	27.8	48.8	59.4	27.8	0.2	0.23
M03	2017	0.22	38	62	62	69.8	38	38	52.2	64.9	38	0.32	0.2
	2018	0.04	14.8	85.2	85.2	56.4	14.8	14.8	38.7	53.8	14.8	0.15	0.25
	2019	0.03	14	86	86	55.5	14	14	38.5	52.7	14	0.05	0.28
	2020	0.05	16.1	83.9	83.9	57.5	16.1	16.1	39.6	54.5	16.1	0.1	0.27
	2021	0.11	25.5	74.5	74.5	62.9	25.5	25.5	45.8	58.4	25.5	0.18	0.24
	2022	0.12	26.9	73.1	73.1	64.5	26.9	26.9	47.6	59	26.9	0.2	0.25
	2023	0.14	28.5	71.5	71.5	65.8	28.5	28.5	48.6	59.8	28.5	0.22	0.23
M21	2017	N/A	N/A	N/A	N/A	N/A	N/A	N/A	N/A	N/A	N/A	N/A	N/A
	2018	N/A	N/A	N/A	N/A	N/A	N/A	N/A	N/A	N/A	N/A	N/A	N/A
	2019	0.01	11	89	89	53.3	11	11	37.4	51	11	0.01	0.4
	2020	−0.06	7.2	92.8	92.8	51.8	7.2	7.2	36.5	49.5	7.2	0	0.42
	2021	0.06	17.5	82.5	82.5	59	17.5	17.5	41.7	55.6	17.5	0.08	0.33
	2022	−0.01	9	91	91	53	9	9	37.1	51.5	9	0.03	0.39
	2023	0.04	14.1	85.9	85.9	56.5	14.1	14.1	39.6	53.5	14.1	0.06	0.36
